# Reduction of *Campylobacter jejuni* in Broiler Chicken by Successive Application of Group II and Group III Phages

**DOI:** 10.1371/journal.pone.0114785

**Published:** 2014-12-09

**Authors:** Jens A. Hammerl, Claudia Jäckel, Thomas Alter, Pawel Janzcyk, Kerstin Stingl, Marie Theres Knüver, Stefan Hertwig

**Affiliations:** 1 Bundesinstitut für Risikobewertung, Berlin, Germany; 2 Freie Universität Berlin, Berlin, Germany; Charité-University Medicine Berlin, Germany

## Abstract

**Background:**

Bacteriophage treatment is a promising tool to reduce *Campylobacter* in chickens. Several studies have been published where group II or group III phages were successfully applied. However, these two groups of phages are different regarding their host ranges and host cell receptors. Therefore, a concerted activity of group II and group III phages might enhance the efficacy of a treatment and decrease the number of resistant bacteria.

**Results:**

In this study we have compared the lytic properties of some group II and group III phages and analysed the suitability of various phages for a reduction of *C. jejuni* in broiler chickens. We show that group II and group III phages exhibit different kinetics of infection. Two group III and one group II phage were selected for animal experiments and administered in different combinations to three groups of chickens, each containing ten birds. While group III phage CP14 alone reduced *Campylobacter* counts by more than 1 log_10_ unit, the concomitant administration of a second group III phage (CP81) did not yield any reduction, probably due to the development of resistance induced by this phage. One group of chickens received phage CP14 and, 24 hours later, group II phage CP68. In this group of animals, *Campylobacter* counts were reduced by more than 3 log_10_ units.

**Conclusion:**

The experiments illustrated that *Campylobacter* phage cocktails have to be carefully composed to achieve the best results.

## Introduction

Campylobacteriosis is a worldwide zoonosis and the most frequent cause of bacterial enteritis in the EU [1]. In the United States campylobacteriosis is estimated to affect more than two million persons every year at great expenses of approximately $1.2 billion annually [2]. Typical symptoms of the disease are diarrhea, cramping, abdominal pain, and fever. Even though a number of pathogenic *Campylobacter* (*C*.) species have been identified, the thermophilic *C. jejuni* and, to a lesser extent, also *C. coli* are responsible for the majority of cases of campylobacterosis [3]. The infection is mainly caused by the consumption and handling of contaminated, undercooked meats, especially poultry [4], [5]. *Campylobacter* is a common commensal of the gastrointestinal tract of various mammals and birds [6]. It is frequently found in chicken farms where the bacteria may spread rapidly [7]. Contaminated birds are shedding up to 10^8^ *Campylobacter* per gram of cecal contents. Hitherto implemented intervention strategies mainly focus on on-farm biosecurity measures and post-slaughter decontamination of poultry carcasses [8], [9]. It has been calculated that a reduction of *Campylobacter* counts on carcasses by two orders of magnitudes could lead to a 30-fold decrease in human campylobacteriosis [10]. However, measures to combat the agent are expensive and not always efficient [11]. Moreover, suitable vaccines have not been made available until now. For these reasons, bacteriophages (phages) have been proposed to reduce the *Campylobacter* counts in chicken [12].

To date several small-scale studies have been performed to evaluate the potential of phages for a reduction of *Campylobacter* in the broiler gut and on chicken skin [9], [13]–[18]. In addition, the first field trial with *Campylobacter* phages (campylophages) has recently been carried out in commercial broiler flocks [19]. In most of these studies, *Campylobacter* counts in cecal content could be reduced by an average of two log_10_ units. In all these studies single phages or cocktails composed of several phages belonging to the same group have been tested. On the basis of their morphology, genome size and endonuclease restriction profile, the currently known lytic *Campylobacter* phages are divided into three groups [20] of which members of group II and group III have yet been used for applications. Group II phages have generally a broader host range than phages of group III since they frequently infect both *C. jejuni* and *C. coli* strains. On the other hand, some group III phages isolated from poultry sources showed a strong lytic activity on certain *C. jejuni* strains [17]. As phage resistance has been perceived as a potential drawback to phage application, it is of great importance that group II and group III phages apparently use multiple host cell receptors for binding [21], [22]. Hence, to overcome the problem of phage resistance in *Campylobacter*, it may be advisable to apply phages belonging to different groups.

In this study, we compared the host ranges and lytic properties of group II and group III phages and used a new approach to achieve a reduction of *C. jejuni* in broiler chicken. The efficacy of a combination of two phages belonging to group II and group III was compared to that of an individual phage. Furthermore, we determined the reduction of *Campylobacter* counts after simultaneous and successive administration of different phages. Finally, resistance frequencies of in vitro- and in vivo-infected bacteria were determined.

## Materials and Methods

### Bacterial strains and growth conditions

Bacterial strains used in this study were obtained from the strain collection of the National Collection of Type Cultures (NCTC, Health Protection Agency, UK) and the National Reference Laboratory for *Campylobacter* of the Federal Institute for Risk Assessment (BfR, Berlin, Germany) (Table 1). Most of the *C. jejuni* and *C. coli* strains originated from the Campynet (CNET) strain collection hosted by the Leibniz Institute DSMZ-German Collection of Microorganisms and Cell Cultures, Braunschweig, Germany, (search term “campynet”). *Campylobacter* spp. strains were stored at −80°C in 10% glycerol stocks. Cultivation of the bacteria was performed on sheep blood agar (blood agar base No. 1, Oxoid, Dassel, Germany) with 5% [vol/vol] defibrinated sheep blood under microaerobic conditions (5% O_2_, 10% CO_2_, and rest N_2_) at 42°C for 24 to 48 h [23]. Further cultivation was performed on Mueller Hinton sheep blood (MH) agar (Oxoid) or in NZCYM broth (Roth, St. Leon-Roth, Germany) [14], [24].

**Table 1:** *Campylobacter* strains and phages used in this study.

Strain	Description	Origin	Reference
*C. jejuni* NCTC11168	Group III phage indicator and propagation strain	Chicken	NCTC [25]
*C. jejuni* NCTC12662	Propagation strain of group II phages CP7 and CP75	Unknown	NCTC [25]
*C. coli* NCTC12668	Group II phage indicator and propagation strain	Unknown	NCTC [25]
*C. jejuni* BfR-CA-3871	Susceptible to group II and -III phages	Sheep (CNET60)	BfR (NRL Campylobacter)
*C. jejuni* BfR-CA-4014	Susceptible to group II and -III phages	Wild bird (CNET28)	BfR (NRL Campylobacter)
**Group II phages**			
CP07	Myovirus, active on *C. jejuni, C. coli*	Faecal sample, poultry, Germany	This work
CP21	Myovirus, active on *C. jejuni, C. coli*	Faecal sample, poultry, Germany	[35]
CP68	Myovirus, active on *C. jejuni, C. coli*	Faecal sample, poultry, Germany	This work
CP75 (NCTC12675)	Myovirus, active on *C. jejuni, C. coli*	UK	NCTC [25]
CP83 (NCTC12683)	Myovirus, active on *C. jejuni, C. coli*	UK	NCTC [25]
CP84 (NCTC12684)	Myovirus, active on *C. jejuni, C. coli*	UK	NCTC [25]
**Group III phages**			
CP1	Myovirus, active on *C. jejuni*	Faecal sample, poultry, Germany	This work
CP14	Myovirus, active on *C. jejuni*	Faecal sample, poultry, Germany	This work
F14	Myovirus, active on *C. jejuni*	Denmark	[24]
CP32	Myovirus, active on *C. jejuni*	UK	[26]
CP81	Myovirus, active on *C. jejuni*	Poultry skin, Germany	[23]

NCTC; National Collection of Type Cultures, Health Protection Agency, UK.

### Isolation, propagation and purification of campylophages

Data on the campylophages used in this study are summarized in Table 1. The majority of the phages (CP1, CP7, CP14, CP21, CP68) was isolated from chicken faecal samples of organic farms in Berlin (Germany) and surroundings. To recover phage particles, faecal samples were suspended in 10 ml SM-buffer (100 mM NaCl, 8 mM MgSO_4_ 7H_2_O, 50 mM Tris-Cl (1 M, pH 7.5)) on a stirrer. Thereafter, the solution was centrifuged at 10,000×g followed by filtration through a 0.2 µm nitrocellulose membrane (VWR International, Darmstadt, Germany). Phage activity was detected by spotting dilutions of the filtrate onto lawns of the *C. jejuni* reference strains NCTC11168, NCTC12662 and *C. coli* NCTC12668 [25].

Some other group II and group III phages of this study have already been described. Group III phage CP81 was recovered from a retail chicken portion purchased from a supermarket in Bavaria (Germany). This phage has been characterized in detail [23]. F14 and CP32 were obtained from phage typing sets of Denmark [24] and the UK [26], respectively. The previously characterized group II phages NCTC12675, NCTC12683 and NCTC12684 obtained from the NCTC were included in the study [25]. All phages were purified by a three-fold recovery of single plaques. High-titre lysates (>10^8^ pfu/ml) of the phages were obtained by infecting 1,000 ml cultures of the host strain (A_600_ of ∼0.3) with phages at a MOI of ∼0.01. After 24 h, lysates were centrifuged for 30 min at 10,000×g to remove debris and then filtrated. Phages were concentrated by ultracentrifugation and purified by CsCl step gradients [27]. Plaque assays, single plaque isolations, and propagation of the phages were performed as previously described [24]. The *C. coli* strain NCTC12668 and *C. jejuni* strain NCTC11168 were used for phage propagation and plaque assays of group II and group III campylophages, respectively [25]. All phages used in this study were analysed by PFGE, restriction analysis and Southern hybridization demonstrating that the members of each group were closely related (data not shown).

### Host range determination

A total of 255 *Campylobacter* spp. strains (*C. jejuni*, 227 isolates; *C. coli*, 18 isolates; *C. lari*, five isolates; *C. fetus*, two isolates; *C. sputorum*, two isolates and *C. hyointestinales*, one isolate) of diverse environmental and clinical origin were used for host range studies. The strains represented more that 20 different MLST types. The host range of the phages was determined by activity assays. 400 µl of the respective indicator strains were mixed with 6 ml prewarmed NZCYM softagar (0.6%) and poured onto a LB agar plate. 10 µl aliquots of the diluted lysates were spotted onto the solid overlay agar. Plates were incubated overnight at 42°C for 24 h under microaerobic conditions [24].

### Stability assays

The stability of the campylophages was investigated at pH values between 4 and 11 and at temperatures between 4°C and 70°C. All stability assays were performed in triplicate.

Phage lysates with adjusted titers of 10^8^ pfu/ml were added to buffer having the respective pH value. The pH of the phage lysate was adjusted at 20°C with glycine hydrochloride (to pH 4.0); citric acid-(di) sodium hydrogen phosphate buffer (to pH 5.0 and 6.0), Tris-HCl (to pH 7.0) or glycine-sodium hydroxide (to pH 8.0 to 11.0). For the test, 900 µl of the adjusted buffer solutions were supplemented with 100 µl of phage lysate, mixed and incubated at 20°C for 24 h and 48 h. Thereafter, aliquots of the buffered lysates were used to determine phage activity. To minimize effects of the pH on the adsorption of phages, only 10^−1^ to 10^−6^ dilutions of the lysates were used for the analysis.

For temperature stability assays, phage lysates (10^8^ pfu/ml) were incubated in a thermomixer at time intervals as indicated. Stability of the phages at −20°C was determined by incubation of lysates (NZCYM broth without cryoprotectant) in a freezer. Thereafter, the phage titer was determined by spotting dilutions onto NZCYM softagar overlay plates.

### Reduction of *Campylobacter* in vitro

For the experiments on in vitro reduction of *Campylobacter*, *C. jejuni* strain BfR-CA-3871 (designated as 3871 throughout this manuscript) was used. Bacteria were cultivated for 24 h as stated above. The cultures were adjusted with NZCYM broth to an adsorption (A_600_) of 0.3 corresponding ∼5×10^7^ cells/ml) and incubated under microaerobic atmosphere. Single phages or cocktails of campylophages were added to the cultures at MOIs (multiplicities of infection) as given in the manuscript. The bacterial titer was determined in time intervals as stated by plating 100 µl aliquots of a ten-fold dilution series on Mueller Hinton sheep blood agar plates.

Based on their in vitro reduction efficacy on *C. jejuni* 3871, two group III (CP14 and CP81) and one group II (CP68) phage were selected for the animal trial (second trial, see below).

### Ethics statement

The animal study (short title: *Campylobacter* reduction) was approved by the local ethic commitee of the state Berlin (Landesamt für Gesundheit und Soziales, Berlin, Germany) under the accession number G 0269/10.

### Animal experiments

For both animal trials, one-day old male Vrolix chicks provided by Aviagen GmbH (Hilbersdorf, Germany) from a *Campylobacter*-free hatchery were transferred to the animal facility of the Federal Institute for Risk Assessment (BfR). After ten days, chickens were randomly allocated to groups according to the study design. It has been reported that transmission of infectious agents between co-housed animals can modify the observed dose-response relationship with implications for the estimation of the infectious dose and the comparison between different agents and treatments [28]. However, in our study the chicken were inoculated with 10^7^ cfu of *Campylobacter*, which much exceeds the minimal infectious dose and led to 100% colonized chicken before phage treatment. Moreover, group housing better reflects real conditions in industrial chicken farms, where coprophagy causes repeated exchange of infectious agents between individual birds. Each group was kept in a room (4.86 m^2^) with a solid floor bedded with wood shavings, provided with red light and a light regime of 13 h day and 11 h night. Feed and water were provided *ad libitum*. Cloacal smears were taken from all animals one week after arrival to confirm that they were *Campylobacter*-free. This was carried out by incubation of the swabs in Preston broth (Oxoid) for 24 h at 42°C under microaerobic conditions. Following this, bacteria were plated on mccDA agar (Oxoid) and incubated for 48 h under the conditions described above. At day 20 of life, the birds were orally inoculated with *C. jejuni*.

In the first animal trial, the colonization efficacy of two selected *C. jejuni* strains (3871 and 4014, Table 1) was determined in two groups (A and B), each of which comprising three birds. Chickens were orally inoculated with 10^7 ^cfu of *C. jejuni* 3871 (group A) and 4014 (group B). The colonization efficacy was determined by *Campylobacter* enumeration in fecal samples after five, six and seven days of infection.

The second animal trial was conducted to determine the potential of three application strategies for the reduction of *C. jejuni* 3871. To ensure that the experiment provides statistically reliable data, four groups (A to D) with ten animals each were analysed. At day 20 of life, all birds were orally inoculated with 10^9^ cfu/ml of *C. jejuni* 3871 suspended in CaCO_3_ to avoid inactivation of the phages by the very low pH of the chicken digestive system [29]. On day 27, the chicken from group B and D received orally a suspension of phage CP14 (5.0×10^8^ pfu), whereas group A remained untreated (control). The chicken of group C received a suspension of the phages CP14 and CP81, each 5.0×10^8^ pfu. On day 28, the animals of group D received a suspension of phage CP68 (5.0×10^10^ pfu).

Fecal samples were collected before phage application and then every 12 h, starting on day 28 (24 h after administration). For this, chickens were separated in cages for 30–60 minutes. When no faeces could be collected, chickens were released and followed until defecation occurred. At day 31, all chickens were killed by decapitation after exposition to electric current. Both ceca were dissected from each animal. During the animal trials, all chickens were observed each day to exclude any unnecessary suffering according to the guidelines of the German law (TierSchG).

### Enumeration of *Campylobacter* and isolation of cells for the determination of phage resistance in vivo

Fecal samples of each chicken were used for the enumeration of *Campylobacter* at different time points during the animal trial. One gram of sample was added to 9 ml of phosphate buffered saline (PBS) and used for the preparation of ten-fold dilutions. 100 µl aliquots of each suspension were plated on Karmali agar containing a *Campylobacter* selective supplement (Oxoid). Plates were incubated at 42°C for 48 h under microaerobic conditions. After incubation, the number of characteristic *Campylobacter* colonies was determined. Ten representative colonies of each chicken were isolated and tested for phage-resistance. Each colony was cultivated in NZCYM broth as stated above, mixed with 5 ml NZCYM softagar (0.6%) and poured on LB agar. Ten-fold dilutions of CP14, CP81 and CP68 lysates were spotted onto the softagar to determine the phage susceptibility of the isolates.

### Statistical analyses

All statistical analyses were performed using the software Stata 13 (StatCorp., College Station, TX, USA). Statistical differences between the application groups of the animal experiments were determined by use of the Wilcoxon Mann-Whitney test, with a Bonferroni corrected alpha level of 0.05/3 = 0.0167 for pairwise comparisons between the control group and the treatment groups (n = 3).

## Results and Discussion

### Selection of phages for the reduction of *C. jejuni*

To select phages that may be suitable for application, eleven group II (n = 6) and group III (n = 5) phages (Table 1) were analysed in terms of their ability to lyse 255 *Campylobacter* spp. strains. In addition, the lytic properties of the phages were determined. As shown in Table 2, all group III phages showed a similar host range by lysing 21 to 26% out of 227 tested *C. jejuni* strains. Other *Campylobacter* species were not infected. Within group II, two subgroups (A and B) disclosing different host specificities were identified. Even though all group II phages were able to infect both *C. jejuni* and *C. coli* strains, subgroup A phages CP68, CP75 and CP84 lysed nearly twice as many strains as the subgroup B phages CP7, CP21 and CP83 (Table 2). Some other authors reported higher percentages of *Campylobacter* strains infected by single phages [14], [15], [17], [24]. However, in none of these studies has such a high number of *Campylobacter* strains been investigated. The obtained data demonstrate the importance of carefully composed phage cocktails. To combat a broad range of *C. jejuni* and *C. coli* strains, cocktails should contain group II as well as group III phages. While group III phages infected higher numbers of *C. jejuni* strains than group II phages, *C. coli* strains were exclusively lysed by phages belonging to group II. In addition, it has to be taken into account that particularly group II phages can be very different with respect to their host specificity. Thus, notably group II phages exhibiting a broader host range (see subgroup A in Table 2) should be used for applications.

**Table 2:** Host range of group II and group III campylophages.

	Group III phages					Group II (A) phages			Group II (B) phages		
	CP1	CP14	F14	CP32	CP81	CP68	CP75	CP84	CP7	CP83	CP21
***Campylobacter*** ** spp. strains** 1	255	255	255	255	255	255	255	255	255	255	255
Positive	56	51	59	50	47	31	30	34	19	15	17
**% positive**	**22.0**	**20.0**	**23.1**	**19.6**	**18.4**	**12.2**	**11.8**	**13.3**	**7.5**	**5.9**	**6.7**
***C. jejuni***	227	227	227	227	227	227	227	227	227	227	227
Positive	56	51	59	50	47	21	20	24	14	13	13
**% positive**	**24.7**	**22.5**	**26.0**	**22.9**	**20.7**	**9.3**	**8.8**	**10.6**	**6.2**	**5.7**	**5.7**
***C. coli***	18	18	18	18	18	18	18	18	18	18	18
Positive	0	0	0	0	0	10	10	10	5	2	4
**% positive**	**0**	**0**	**0**	**0**	**0**	**55.6**	**55.6**	**55.6**	**27.8**	**11.1**	**22.2**

1 None of the other *Campylobacter* species: *C. lari* (n = 5), *C. fetus* (n = 2), *C. sputorum* (n = 2) and *C. hyointestinales* (n = 1) was lysed by the phages.

**Figure 1:**
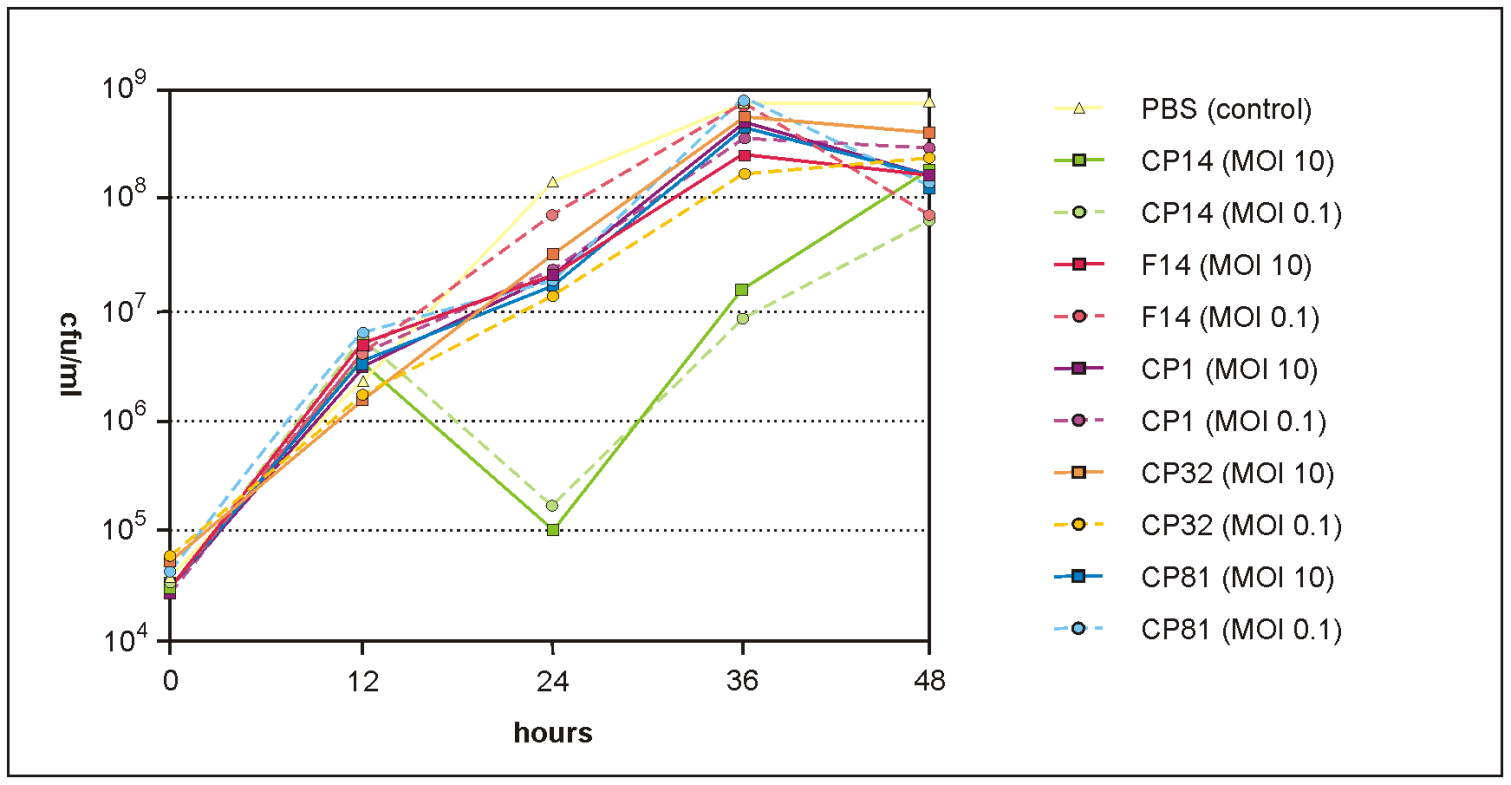
Reduction of *C. jejuni* 3871 by group III phages in vitro. Each experiment was performed in triplicate. For better clarity, only mean values without standard deviations are shown.

Infection of *C. jejuni* under in vitro conditions revealed a diverse lytic potential of the investigated group III phages. Compared with the non-phage-treated control, CP14 most efficiently (by approximately 3 log_10_ units) reduced the *C. jejuni* 3871 counts at both applied MOIs of 0.1 and 10 after 24 h of incubation (Fig. 1), whereas a reduction of only 1 log_10_ unit was achieved with the remaining group III phages. A combined application of CP14 with other group III phages (e.g. CP81) did not result in higher reductions (data not shown). The group II (A) phages CP68, CP75 and CP84 showed very similar reduction rates and there were no striking differences discernible at different MOIs or after (simultaneous or successive) infection of the bacteria with two phages. In general, group II phages caused slightly lower reductions than group III phages. However, cell lysis occurred earlier (after 9 h) and remained constant for at least further 15 hours. The data on group II and group III-induced cell lysis are in good agreement with quantitative models of in vitro bacteriophage-host dynamics created by Cairns et al. (2009) [30]. Similar to the published results obtained with group III phage CP8, no significant differences in the kinetics of cell lysis were observed with our phages at MOIs of 0.1 and 10. Using an initial cell concentration of 5×10^4^ cfu/ml, the required proliferation threshold of bacteria was exceeded while the inundation threshold of phages was at maximum 5×10^3^ pfu/ml (Fig. 1).

**Figure 2:**
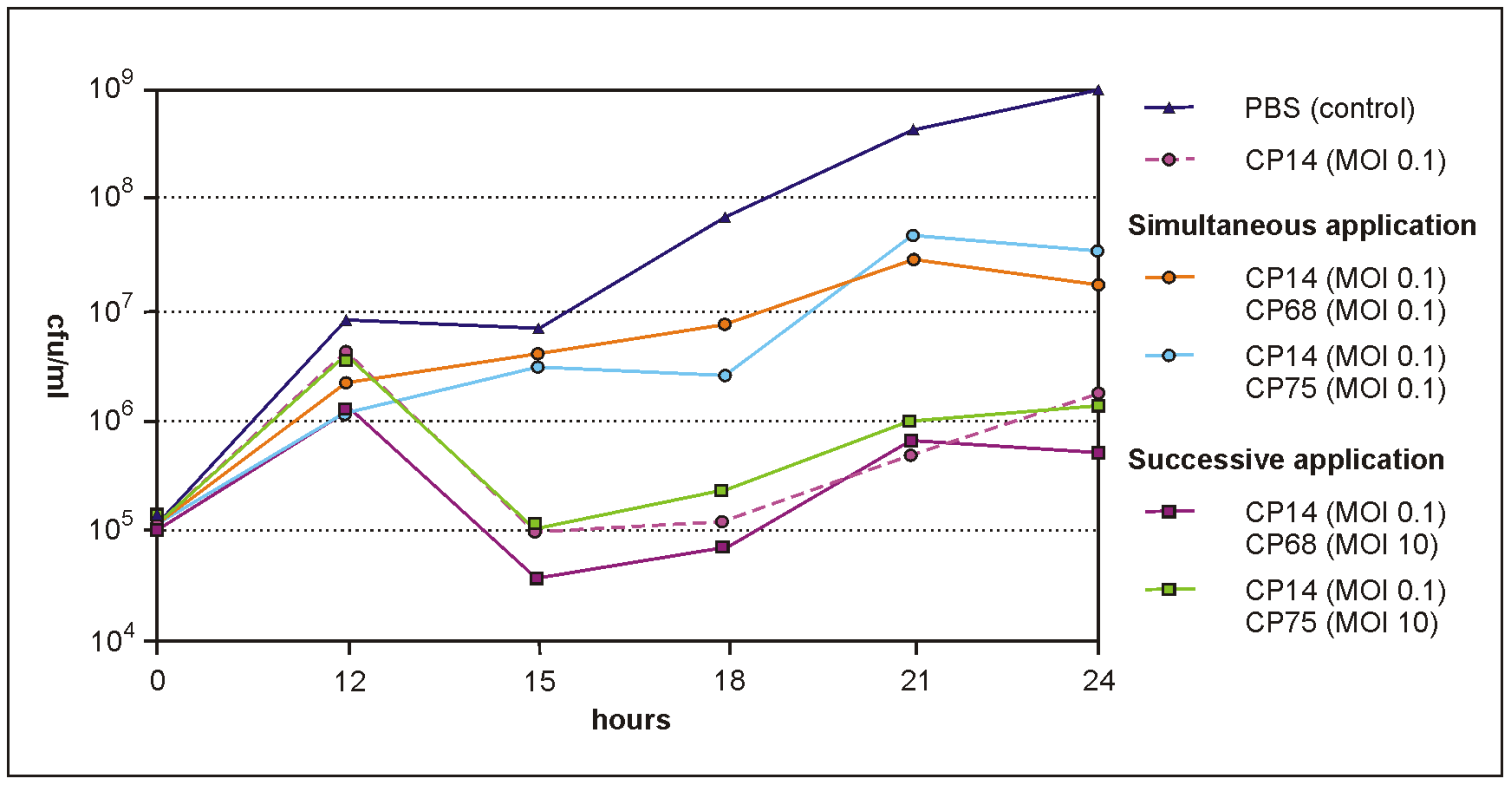
Reduction of *C. jejuni* 3871 in vitro by simultaneous and successive application of group II and group III phages. Each experiment was performed in triplicate. For better clarity, only mean values without standard deviations are shown.

In the last set of in vitro experiments, we compared CP14-induced *C. jejuni* 3871 cell lysis with the lytic activity of a combination of CP14 with a group II phage (CP68 or CP75). Since group II phages cleared the host cell culture significantly faster than group III phages (see above), they might be better suited for a reduction of the bacteria shortly before slaughtering. For that reason, the phages were added to the culture successively by adding the respective group II phage at a high MOI of 10 18 h later than CP14. Similar to the experiments before, CP14 lysed the bacteria efficiently (Fig. 2). The strongest reduction, related to the control culture, was observed after 18 to 24 h of incubation. However, while in the presence of CP14 alone, the cell number increased again by approximately 1.3 log_10_ units between these time points, the addition of group II phage CP68 resulted in an approximately 0.5 log_10_ units lower cell number after 24 h (Fig. 2). By contrast, neither a cocktail composed of CP14 and CP75 nor the simultaneous application of CP14 and CP68 led to a higher reduction than those achieved with CP14 alone. The reason for this result remains unknown. While phage-induced lysis of *Campylobacter* under in vitro conditions has already been reported by other authors [31], [32], this is the first study in which the kinetics of host cell lysis induced by group II and group III phages has been compared. Moreover, a combined action of a group II and a group III phage has not been demonstrated before. The obtained data indicate that it might be conducive to use low numbers of group III phages for longer incubation times allowing phage replication while group II phages applied at high MOIs might be better suited for “lysis from without”, the phenomenon of lysis caused by adsorption of numerous phages to each cell. To elucidate whether the period needed for DNA replication, packaging and virion assembly or the number of released particles account for the different lysis times, the latent period and burst size of some of the phages was determined. All of them revealed a similar latent period between 67 and 82 min, which corresponds well with data determined for some other group II phages [14]. Though, the burst size of group II phage CP68 was more than twice as large (14 to 20 pfu) than those of the group III phages CP14 (6 to 7 pfu) and CP81 (4 to 6 pfu) (data not shown). Values between 9 and 24 pfu have also been reported for group II phages in the study cited above. The data suggest that the fast lysis of bacterial cultures induced by group II phages is mainly caused by their high burst size.

### Stability of the phages

**Figure 3:**
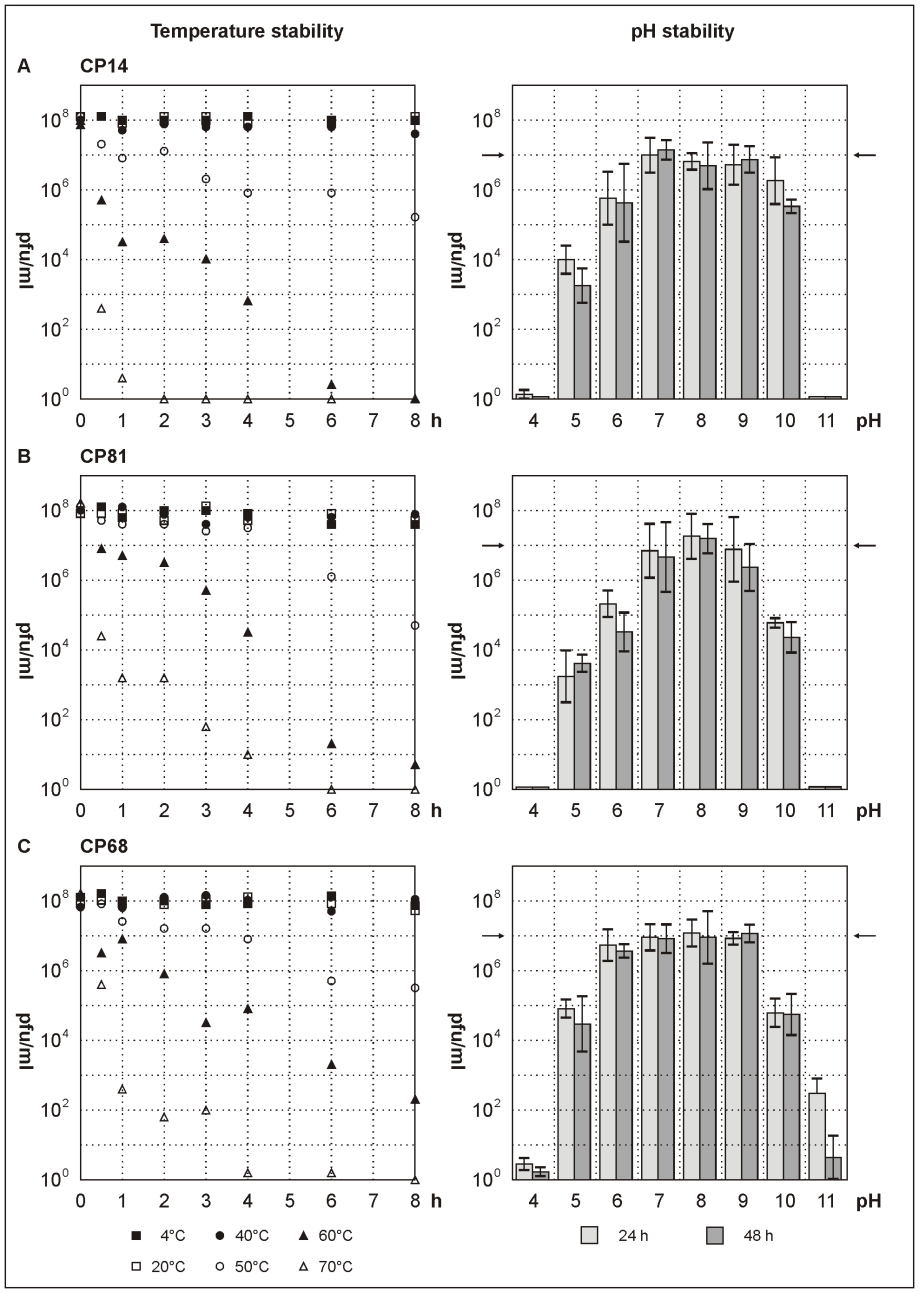
pH and temperature stability of the phages CP14, CP81 and CP68. Each experiment was performed in triplicate. Bars indicate standard deviations. pH stability was tested at 20°C. As indicator strain for phage activity (quantified as PFU/ml), *C. jejuni* 3871 was used. Arrows indicate the initial phage titers.

Incubation of the eleven tested phages at temperatures between 4°C and 70°C for 8 h revealed that they are similarly stable. At temperatures up to 40°C, the phages remained fully active while higher temperatures caused a gradual loss of activity (Fig. 3). The long-term stability of the phages was studied for up to three years. Storage at 4°C and 25°C resulted in a decrease of activity by approximately 1 log_10_ unit after one year and by two to three log_10_ units at the end of the experiment (after three years). Freezing at −20°C decreased the phage titer by four and seven log_10_ units after one and two years of incubation, respectively, whereas no activity was detectable after three years.

The phages remained fully active for at least 2 h at pH values between pH 5 and pH 9 (data not shown) and for at least 48 h at pH values between pH 7 and pH 9 at room temperature (Fig. 3). For group II phage CP220, an extended pH stability has been reported [15]. To ensure protection from the low pH of the chicken digestive system, the birds were fed with 1 ml of 30% CaCO_3_ solution before phages were administered.

### Animal experiments

In the first experiment (pre-trial), the capability of two *C. jejuni* strains (3871 and 4014, Table 1) to colonize the chicken gut was investigated (see Material and Methods). The strains had previously shown sensitivity to group II and group III phages under in vitro conditions. In addition, the in vivo stability of phages in the absence of *Campylobacter* was studied after administration to two other groups of animals (data not shown). Both *Campylobacter* strains colonized the chickens efficiently. After administration of 10^7^ cfu to 20 day-old birds, bacteria were identified in excreta of all animals. While cell numbers between 3 and 5.3 log_10_ cfu/g of intestinal content were determined at 48 h, the numbers increased up to 7.7 log_10_ cfu/g at day 26. On day 27, the chickens were killed and *Campylobacter* counts between 7.6 and 8.5 log_10_ cfu/g were determined in the caecum. Strain 3871 generally showed slightly higher rates of colonization than strain 4014 and was therefore selected for the phage treatment. Unlike the bacterial strains, the administered phages could not be identified in excreta and in the caecum of the birds. It can be surmised that the number of applied phages (10^7^ pfu) was too low to detect them. Moreover, phage propagation within the chicken intestine could not occur due to the absence of a suitable host. A strong decrease of phage titer by several orders of magnitude has also been observed in an experiment where 10^10^ phages were administered to *Campylobacter*-free chickens [18].

**Figure 4:**
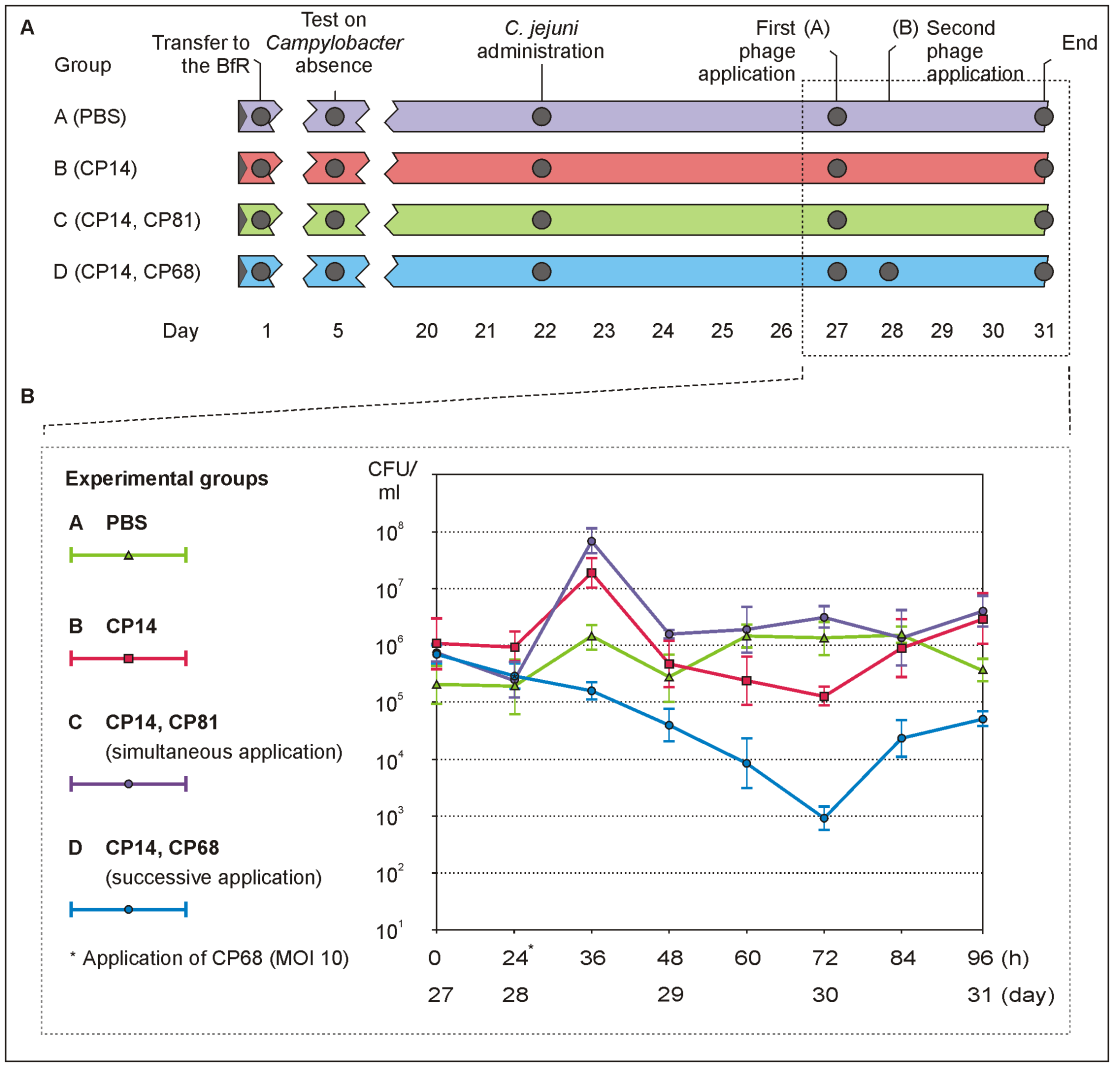
Design and outcome of the animal experiment. A. Time scale of the experiment. B. Reduction of *C. jejuni* in the chickens. Mean values of *Campylobacter* counts (CFU/g feces) and standard deviations are shown.

Based on the in vitro data and the results achieved in the pre-trial, the main animal experiment was designed. The following questions should be answered (i) Is a simultaneous application of two group III phages more efficient than the administration of a single (group III) phage? (ii) How is the efficacy of a successive application of a group III and a group II phage? To answer these questions, *C. jejuni* strain 3871 (10^7^ cfu) was administered to four groups (A to D) of 20 days old chickens (A: 9 animals, B to D: 10 animals each) (Fig. 4A). On day 27 group B and group D received a dose of phage CP14 (MOI 0.1) while group C got a cocktail comprising the phages CP14 and CP81, both of which administered at a MOI of 0.1. Even though this cocktail did not perform better than CP14 alone under in vitro conditions, the combined activity of two phages might be beneficial in the chicken gut. On day 28, group D received an additional dose of phage CP68 at a MOI of 10. The last group (A) of animals served as control and did not get any phage. From day 27 to day 31 excreta of the birds were collected in time intervals of 12 h and *Campylobacter* and phage counts were determined. On day 31 all chickens were killed and caecal contents were analysed. Fig. 4 summarizes the results on *Campylobacter* reduction. The data show that, compared to the control, group III phage CP14 (experimental group B) caused a statistically significant reduction from 48 h onwards with a maximal *Campylobacter* decline (more than one log_10_ unit) after 72 h (p = 0.0137). In some other studies with group III phages, the highest reduction was already observed after 24 to 48 h [14], [15], [17], [18]. However, the collected data can be hardly compared since the numbers of administered bacteria, phages and the resulting MOIs were not the same. The concurrent administration of a second group III phage (CP81, experimental group B) did not yield a significant reduction of *Campylobacter* (p = 0.121). It is conceivable that there was a yet unknown interference between CP14 and CP81 which inhibited phage propagation or that CP81 induced resistance which also affected CP14 (see below). In the in vitro experiments a cocktail of both phages significantly reduced *Campylobacter* counts by up to 3 log_10_ units (data not shown). Hence it remains open why no reduction was attained in chickens. Consistently, Wagenaar et al. (2005) reported that an application of two group III phages led to a similar reduction (1.5 log_10_ units) of *C. jejuni* as a single phage [18]. The authors could not detect any antagonistic effect under in vitro conditions.

In our study the by far highest reduction was achieved by a combined action of group III phage CP14 and group II phage CP68 that had been administered successively (Fig. 4B). 48 h after application of the second phage CP68, a more than 3 log_10_ units lower number of *Campylobacter* was determined compared to the untreated control (p = 0.0111). Even though bacterial counts increased again until the end of the experiment, they remained significantly lower than in the control group and in group B which had been exclusively treated with CP14. We suspect that the fast cell lysis and high burst size of CP68 in conjunction with its from CP14 divergent host cell receptor were mainly responsible for the increased lytic activity observed in experimental group D.

### Phage resistance of *C. jejuni* 3871 in vivo and in vitro

As resistance is of great importance for the outcome of phage application, we analysed both the susceptibility of the bacteria under in vivo and under in vitro conditions. Therefore, ten colonies isolated from the caecum of each bird were analysed in terms of their susceptibility to CP14, CP81 and CP68. Table 3 illustrates that resistance of all isolates against group II phage CP68 was rather low with frequencies between 1.0 and 2.0%. By contrast, the group III phages provoked higher rates of resistance against themselves. Most notably, frequencies of 7 and 8% were determined in group C, in which both group III phages were applied. As the administration of CP14 alone (group B) yielded resistance frequencies of only 5% (CP14) and 4% (CP81), it might be concluded that the elevated levels of resistance observed in group C were mainly caused by CP81. This could explain why *Campylobacter* counts were not reduced in group C. The in vivo data on phage resistance were corroborated by in vitro experiments (see Materials and Methods). Colonies that were resistant to a particular phage showed also resistance to all other phages of the same group but not to phages belonging to the other group. In addition, resistance to group II phages was much more stable than resistance to phages of group III, which in some cases quickly reverted to susceptibility, as already reported by Fischer et al. (2013) and Scott et al. (2007) [33], [34]. Reversion to phage susceptibility can be caused by phase variable capsular polysaccharide structures [22]. Even though several group III phages investigated in the cited study were highly specific in the recognition of a particular combination of capsular modifications, they caused cross-resistance. Thus, it is advantageous to analyse resistance of all phages of a cocktail thoroughly and to compose phages belonging to different groups, which differ in their host ranges, lytic properties and resistance mechanisms.

**Table 3:** Resistance frequencies in vivo.

Experimental groups	No. of isolates tested (n)^1^	Resistance to CP14^2^	Resistance to CP81^2^	Resistance to CP68^2^
**A (PBS control)**	90^3^	2.2% (88)	2.2% (88)	1.1% (89)
**B (CP14)**	100	5.0% (95)	4.0% (96)	2.0% (98)
**C (CP14, CP81)**	100	7.0% (93)	8.0% (92)	1.0% (99)
**D (CP14, CP68)**	100	5.0% (95)	3.0% (97)	2.0% (98)

1 Ten isolates of each chicken were investigated.

2 The number of phage susceptible isolates is given in brackets.

3 One chicken died during the adaption phase.
